# Adherence in HIV-positive patients treated with single-tablet regimens and multi-pill regimens: findings from the COMPACT study

**DOI:** 10.7448/IAS.15.6.18098

**Published:** 2012-11-11

**Authors:** A Antinori, C Angeletti, A Ammassari, D Sangiorgi, A Giannetti, S Buda, E Girardi, L Degli Esposti

**Affiliations:** 1 National Institute for Infectious Diseases, Clinical Department, Rome, Italy; 2 National Institute for Infectious Diseases, Epidemiological Department, Rome, Italy; 3 CliCon S.r.l., Ravenna, Italy

## Abstract

The use of Combination AntiRetroviral Therapy (cART) has decreased the morbidity and mortality of patients infected with HIV. However, adherence to cART remains crucial to prevent virological failure and disease progression. The aim of this study was to assess adherence to treatment among patients treated with Single Tablet Regimen (STR) or with multi-pill regimens based on Protease Inhibitors (PI), Non-Nucleoside Reverse-Transcriptase Inhibitors (NNRTI), or raltegravir (RAL). An observational retrospective cohort analysis based on administrative and clinical databases was conducted at the National Institute for Infectious Diseases (Rome, Italy). HIV-positive patients treated with a cART between Jan 1st, 2008–Dec 31st, 2010 were included. Patients were followed-up for one year since the first prescription during the inclusion period or up to death or switch of at least one drug of the regimen. Adherence and selective non-adherence (days without backbone or 3rd drug) were calculated using pharmacy refill compliance [1]. cART regimens were classified based on number of daily pills (STR vs multi-pill regimen) and on type of third drug. Viral Load (VL) and CD4 cell counts at the end of the follow-up were evaluated. A total of 1,604 patients were analyzed, 70.0% male, age 45.0±8.7, 14.3% newly treated. Patients on STR were 159 (9.9%), PI 878 (54.7%), NNRTI 523 (32.6%), RAL 44 (2.7%). Presence of at least one AIDS-defining conditions (according to Centers for Disease Control classification) was 30% in the STR group, 34% PI, 26% NNRTI, 34% RAL (p=n.s.). Adherence was 80.4±14.7% for STR, 71.8±21.8% PI, 77.1±20.3% NNRTI, 74.0±22.4% RAL. Selective non-adherence was 5.5% (18 days) PI, 2.8% (8 days) NNRTI, 12.5% (43 days) RAL (Figure 1). At the end of the follow-up, VL/CD4 values were available among 709 patients (44%); CD4 count >500 cell/mm3 was observed among 61% of patients on STR, 44% PI, 48% NNRTI, 42% RAL and VL < 50 copies/ml was observed among 96% of patients on STR, 78% PI, 88% NNRTI, 87% RAL. Interruptions in cART refill remain a relevant problem across all cART regimens. Patients on STR displayed a higher adherence rate compared to multi-pill regimes (PI, NNRTI, and RAL), primarily due to lack of selective non-adherence. Patients on STR experienced also higher rates of VL < 50 and CD4 > 500. The use of an STR regimen appears an effective therapeutic option to avoid selective non-adherence and, consequently, to prevent virological failure and disease progression.

**Figure 1:**
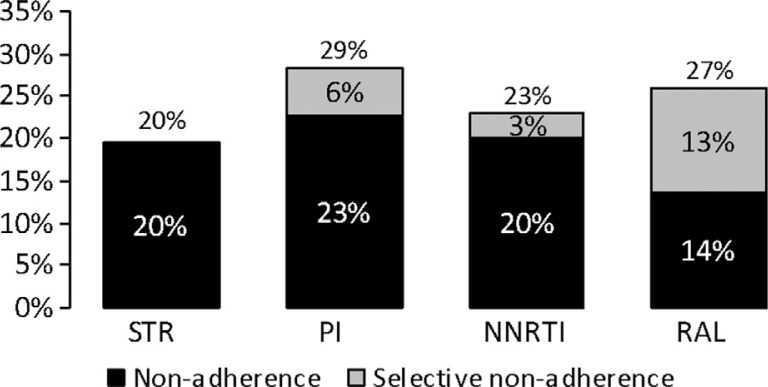
Non-adherence to cART regimens.

Non-adherence to cART regimens.
